# Commentary on: “Targeting fibrotic scarring by mechanoregulation of Il11ra1^+^/Itga11^+^ fibroblast patterning promotes axon growth after spinal cord injury”

**DOI:** 10.4103/NRR.NRR-D-25-01676

**Published:** 2025-11-25

**Authors:** Kwok-Fai So

**Affiliations:** Key Laboratory of CNS Regeneration (Ministry of Education), Guangdong-Hong Kong-Macau Institute of CNS Regeneration, Jinan University, Guangzhou, Guangdong Province, China; State Key Laboratory of Brain and Cognitive Science, Li Ka Shing Faculty of Medicine, The University of Hong Kong, Hong Kong Special Administrative Region, China

The fibrotic scar due to excessive deposition of extracellular matrix (ECM) after spinal cord injury (SCI) remains one of formidable challenges to axonal regeneration. Previous therapeutic strategies mainly focus on eliminating fibrotic scars by blocking (Göritz et al., 2011) or inhibiting (Dias et al., 2018) the generation of scar-forming stromal cells, as well as inducing their migratory defect (Hellal et al., 2011; Ruschel et al., 2015). Although these approaches help reduce fibrotic scarring, it is insufficient to fully reverse the pathological consequences of fibrosis. Increasing evidence shows that fibrotic scars actually play important positive roles by sealing the lesion and preserving tissue integrity. Moreover, interactions between regenerating axons and ECM are indispensable for axonal elongation and growth (Anderson et al., 2018). This dual nature of healthy physiological ECM and pathological scars means that eliminating the fibrotic scar is not ideal for SCI repair.

In a recent *Science Advances* publication, Tan et al. (2024) demonstrated that an integrating hydrogel composed of hyaluronic acid-graft-dopamine (HADA) and a designer peptide HGF-(RADA)4-DGDRGDS (HRR) could reprogram fibrotic responses after SCI. Rather than blocking scar formation, the HADA/HRR hydrogel guided the organized infiltration of platelet-derived growth factor receptor β-positive fibroblasts into SCI lesions, creating a parallel, axon-permissive ECM that guides axonal regrowth. This pioneering strategy focused on modulating primary cellular responses rather than eliminating dense scars, proposing a revolutionary innovation in fibrotic scar therapy.

Following this seminal discovery, the research group of He et al. expanded the series in *Advanced Science* by dissecting the mechanistic basis of this regenerative fibrotic remodeling (Xiao et al., 2025). They demonstrated that the HADA/HRR hydrogel with the stiffness matching that of native spinal cord, referred to as “Medium,” induced fibroblast alignment and reshaped fibrotic scars into a parallel matrix, while the stiffness deviating from that of spinal cord fails to do so. This elegant continuation not only validated axonal regeneration, but also deepened it mechanistically by demonstrating that mechanotransduction acts as the decisive cue driving ordered fibroblast behavior.

Utilizing single-cell RNA sequencing, they identified a previously unknown Il11ra1^+^/Itga11^+^ fibroblast subset associated with the ordered infiltration. *In vitro* and *in vivo* investigations both indicated that these cells showed polarized phenotypes when migrating into HADA/HRR Medium hydrogels, characterized by cytoskeletal alignment with anisotropic adhesion forces and elevated DetyrTub expression. In contrast, cells displayed isotropic patterns on non-optimal stiffnesses. Furthermore, the authors delineated a distinct mechanosensitive signaling cascade. In this process, the LRP6 receptor on fibroblast surface was dephosphorylated, and consequently downregulated matrix metalloproteinase-7 (MMP-7) expression through the LRP6/β-Catenin/MMP7 signaling pathway. The parallel ECM guided axonal regrowth, promoting neural reconnections across the lesion.

Together, the series of studies, spanning from discovery of ECM-guided regeneration to mechanistic elucidation of fibroblast mechano-responsiveness, forms a coherent narrative that redefines fibrotic scar biology as a tunable and regenerative microenvironment. A revolutionized treatment of fibrotic scars is proposed by targeting key cellular behaviors that drive scar formation. Such conceptual innovation transforms pathological scars into healthy physiological ECM, revealing a new principle for precision tissue engineering and regenerative therapeutics. Beyond SCI, this framework provides a mechanistic blueprint for modulating fibroblast behavior in fibrotic organs with excessive extracellular matrices, potentially allowing organ-specific ECM remodeling. Coupling with advanced biomaterials and mechano-sensitive signaling modulation, these strategies could integrate cellular, molecular, and structural levels to optimize tissue repair outcomes. Systematic evaluation in large animal models, including functional readouts and safety assessments, will be pivotal to translate these insights into clinical interventions, potentially establishing a generalizable platform for antifibrotic therapy across diverse tissues.

*The author KFS is the Editor-in-Chief of Neural Regeneration Research. He was blinded from reviewing or making decisions on the manuscript.*
